# High-Resolution Laser Interference Ablation and Amorphization of Silicon

**DOI:** 10.3390/nano13152240

**Published:** 2023-08-03

**Authors:** Andreas Blumenstein, Peter Simon, Jürgen Ihlemann

**Affiliations:** Institut für Nanophotonik Göttingen e.V., Hans-Adolf-Krebs-Weg 1, 37077 Göttingen, Germany

**Keywords:** UV femtosecond laser, laser interference patterning, silicon, amorphization, laser-induced periodic surface structures

## Abstract

The laser interference patterning of a silicon surface via UV femtosecond pulse irradiation, resulting in 350 nm periodic structures, is demonstrated. The structuring process was performed using a laser with a 450 fs pulse duration at a wavelength of 248 nm in combination with a mask projection setup. Depending on the laser fluence, single-pulse irradiation leads to amorphization, structure formation via lateral melt flow or the formation of voids via peculiar melt coalescence. Through multipulse irradiation, combined patterns of interference structures and laser-induced periodic surface structures (LIPSS) are observed.

## 1. Introduction

The laser generation of periodic surface structures has developed into a broad field with many different techniques and a range of potential applications like microlasers, optical nanoswitches or storage devices, sensors or antifraud features [1]. Such structures can modify the tribological characteristics [2], wettability [3] or cell growth properties [4] of surfaces. For the fabrication of micron- or sub-micron-pitch structures, especially on metals and semiconductors, mainly ultrashort pulse lasers are applied.

An important figure of merit, the heat-affected zone (HAZ) can be kept small by ensuring rapid energy deposition. This can be achieved by applying ultrashort pulses, making very high spatial resolution feasible for virtually any material. However, the topography of the resulting pattern depends strongly on the underlying physical processes and can either show a similar shape resembling the spatial distribution of the irradiation, or may differ from it substantially [5]. The parameters governing the involved processes include the photon energy, the pulse length, the number of pulses hitting the surface, the material’s band structure and the concentration of the free carriers [6]. Phenomenological processes like evaporation, melting or hydrodynamic motion will be driven by these parameters. In the case of metals, for example, the free carriers are responsible for the absorption of the incident photons. Afterwards, the energy transfer to the lattice follows within a few picoseconds, determined by the electron–phonon relaxation time of the material. As a consequence, a further reduction in the pulse duration below the electron–phonon relaxation time would not cause any change in the result [7]. Semiconductors like silicon behave very similarly to metals in these respects. A high density of free carriers is generated very fast here during the ultrashort laser pulse [8].

Most investigations on periodic patterning deal with so-called laser-induced periodic surface structures (LIPSS) that develop after single-beam and mostly multipulse irradiation within a certain laser fluence range [9]. For silicon, a number of works have been conducted in which the authors generate LIPSS to understand their formation process, such as in [9,10] and references therein. Using fs lasers with wavelengths typically in the range of 800 to 1100 nm, structure periods are normally in the µm range. Only so-called high-spatial-frequency LIPSS (HSFLs) exhibit periods of a few hundred nanometers [11,12]. On silicon, such HSFLs have been observed with periods of 125 nm and 110 nm after multiple-pulse irradiation [13,14]. 

A method capable of generating deterministic periodic patterns is interference ablation [15]. Overlapping at least two coherent beams, an interference pattern is obtained with a spatially modulated intensity distribution. If the peak intensity is high enough to create a permanent surface modification, a periodic relief pattern will be created. In these schemes, usually, amplitude or phase gratings are used to split a beam into two or more partial beams, which are then recombined on the surface of the target. By manipulating the amplitude, the phase and the polarization of the interfering beams, various intensity distribution can be obtained. The applied wavelength and the angle of interference will determine the achievable minimum feature size. The irradiation parameters, the material properties and the specific environmental conditions are responsible for the resulting structure details. Independent of the beam management, the ablation results will strongly depend on the laser pulse characteristics. At the same interference angle, UV beams generate smaller periods compared to visible or IR beams. Furthermore, for many materials, the optical absorption is stronger in UV compared to longer wavelengths, leading generally to better ablation quality. This is especially the case for widely transparent materials like glasses. However, not only is the wavelength of great importance, but so is the pulse duration. If the thermal conductivity of the work piece material is high, as it is, for example, in the case of metals or semiconductors, shorter pulses lead to smaller heat-affected zones, and therefore, higher contrast when reducing the structure period. For sub-µm patterns on metals, only ultrashort (ps or fs) pulses are suitable for obtaining satisfying results. A further benefit of using ultrashort pulses is their capability of inducing multiphoton absorption, so that even transparent materials can be treated with visible or IR fs pulses. An example is the fabrication of periodic patterns via two-photon polymerization utilizing multibeam interference [16].

Beam interference concepts have been applied to create deterministic patterns with a predefined period and control of the surface profile [17]. For moderate period sizes in the range of a few µm to tens of µm, mostly nanosecond pulsed lasers are applied [18,19]. If periods in the µm or sub-µm range are desired, especially in case of metals and semiconductors, ultrashort pulses of picosecond or femtosecond duration are used [20,21,22]. However, it has been observed, in many cases, that the local ablation depth does not correlate with the local laser fluence in a simple manner as could be expected from large-area ablation experiments, where a monotonously increasing ablation depth with increasing fluence is observed. Instead, the formation of special features like bumps, voids, ridges and droplets is observed, indicating that on this scale, other contributions like lateral material movement have to be considered [23].

The smallest structure period obtained on silicon to date via two-beam interference patterning with IR-fs pulses (560 fs/1030 nm) is 720 nm [24]. In a low-fluence regime, swelling of the material is observed along the lines of highest intensity. At higher fluence values, grooves are generated, with ridges on both sides elevating to a typical height of a few nanometers. Possible explanations for the development of these protrusions could be material expansion either upon re-solidification or caused by oxidation of the overheated silicon.

In order to obtain higher-spatial-frequency structures using interference ablation, a shorter wavelength, preferably in the UV range, has to be applied. This way, patterns with a period of 300–400 nm have been generated on various metals and semiconductors [20]. Whereas the structure formation in the case of gold has been studied in detail [5,6], no such study has been available for silicon until now. In this paper, we investigate this structure formation via scanning electron microscopy (SEM), atomic force microscopy (AFM) and transmission electron microscopy (TEM) at various laser fluences, and show that deterministic relief patterns with periods of 350 nm and deterministic structure details like voids 30 nm in size can be generated.

Besides the generation of relief patterns, the laser-induced amorphization of silicon is of interest [25]. Laser writing of amorphous Si structures could be used for creating lines of higher refractive index than the crystalline phase of the standard Si wafer to be applied as waveguides in photonic integrated circuits. LIPSS in the form of phase change patterns have been intensively studied [26,27]. Deterministic amorphization patterns with high spatial resolution will be investigated as well in this paper.

## 2. Materials and Methods

**Figure 1:**
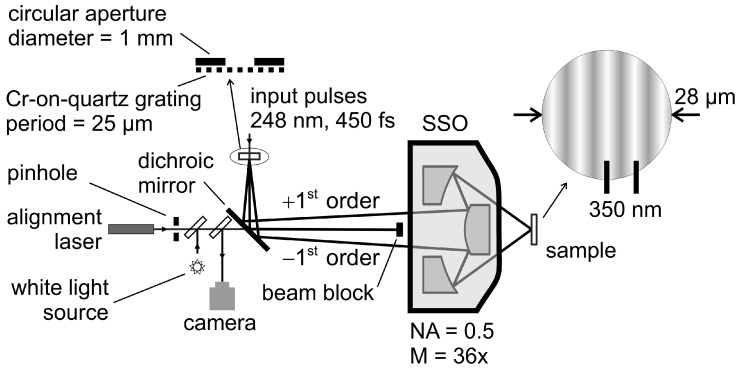
Scheme of the optical setup.

The experimental setup is described in detail in [6]. Frequency-tripled Ti:Sa laser pulses were used to seed a KrF excimer amplifier, producing pulses with energies of up to 30 mJ at a wavelength of 248 nm and a repetition rate of 10 Hz. The pulse duration of 450 fs was measured by using a frequency-resolved optical gating (FROG) device. Single pulses out of the pulse sequence were selected using a mechanical shutter. The used pulse energy was set using a variable attenuator. Single-pulse exposure using a combination of two-beam interference and mask projection led to a processed area on the sample surface with a sinusoidal intensity distribution with a periodicity of 350 nm (Figure 1). The central part of the laser beam with a flat top distribution illuminated the mask, comprising a circular aperture of 1 mm diameter in contact with a Cr-on-quartz grating of lines and spaces with a 25 µm period and a duty cycle of 0.5. For mask projection, a Schwarzschild-type objective with a numerical aperture (NA) of 0.5 and a demagnification (M) of 36× was used. The Schwarzschild objective (SSO) had a highly reflective (HR) dielectric coating for 248 nm (manufactured by Ealing).

The benefits of mirror-based objectives compared to lens systems are as follows:

- They have superior transmission and damage threshold based on high-reflectivity dielectric coatings, providing a particular advantage in the UV range, where multi-photon absorption in transparent materials is usually critical.
- Because of the lack of refractive materials, they are free of chromatic aberration.
- Providing the same amount of numerical aperture, their working distance is larger compared to refractive objectives, facilitating improved target handling and ensuring less debris deposition on the optical surfaces.

Based on the aperture size in the mask plane and the demagnification of the objective, the diameter of the overall projected area on the workpiece was 28 µm. Since all diffracted orders emerging from the transmission grating (placed in the mask plane), except the two first order beams, were blocked, the image was formed via two-beam interference, resulting in a sinusoidal intensity distribution across the 28 µm image area. The spatial frequency of this modulation was doubled by blocking the zeroth order diffraction using a beam block placed in front of the SSO, thus resulting in a period of 350 nm on the workpiece (instead of a pitch size of 700 nm based on the nominal demagnification). 

Special care had to be taken when positioning the sample precisely in the image plane. This was rather critical due to the short overlapping length of the interfering beams resulting from the large angle between them and because of the short Rayleigh-length of the focusing caused by the short focal length of the SSO. For the sample positioning, a separate beam path over a dichroic mirror (HR at a 248 nm and transparent in the entire visible spectrum) was set up. In this beam path, a pinhole with a diameter of 35 µm was placed, which was illuminated by an alignment laser operating at 532 nm. The position of the pinhole was adjusted in such a way that both the pinhole and the grating in the mask plane had the same distance to the SSO and were therefore imaged simultaneously onto the workpiece. Diffraction of the alignment laser beam on the pinhole ensured that most of the diverging beam passed the beam block and entered the SSO. In the beam path of the alignment pinhole, two additional beam splitters were mounted for sample observation. Over one of them, a white light illumination was coupled into the beam path, and the other was used to couple the beam, returning from the sample to a camera for live observation of the sample surface. In the irradiation experiments, polished Si wafers were used.

The surface analysis of the samples after irradiation was performed via scanning electron microscopy (SEM; Zeiss EVO MA10). The images were recorded at a high voltage of 15 kV using a secondary electron detector. The conductivity of silicon was sufficient to investigate the samples without additional gold coating. The surface topography of the samples was measured via atomic force microscopy (AFM; Park Systems, XE-150 with ACTA-10 non-contact cantilever) and via transmission electron microscopy (TEM) using a 300 kV FEI Titan G2 ETEM. The samples were prepared via focused ion beam thinning (FEI Helios FIB) of the cross section lamellas, and thus, coated with polycrystalline Pt in order to protect the surface during milling.

## 3. Results and Discussion

**Figure 2:**
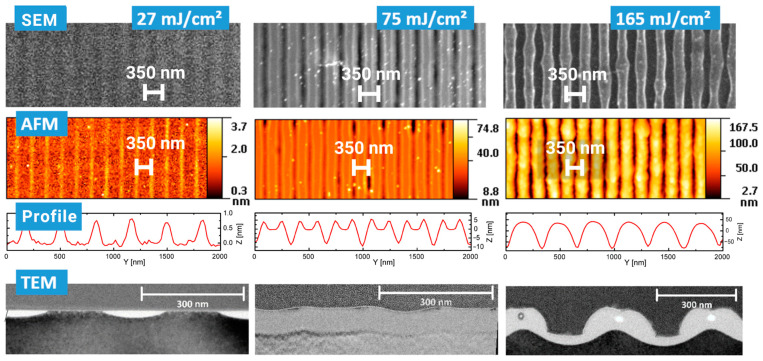
Structure formation on silicon under variation in the (average) laser fluence. SEM (**top**), AFM (**middle**) and TEM (**bottom**) records are displayed.

**Figure 3:**
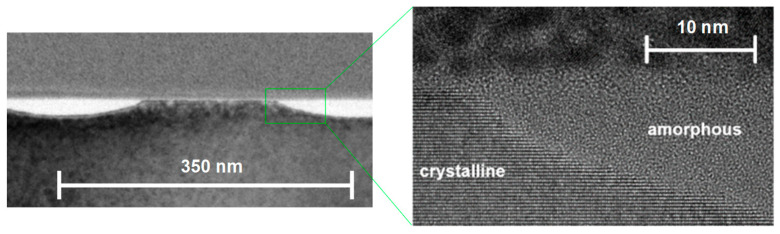
Close-up of the TEM cross section at an average fluence of 27 mJ/cm^2^. Crystalline and amorphous regions can be clearly distinguished.

**Figure 4:**
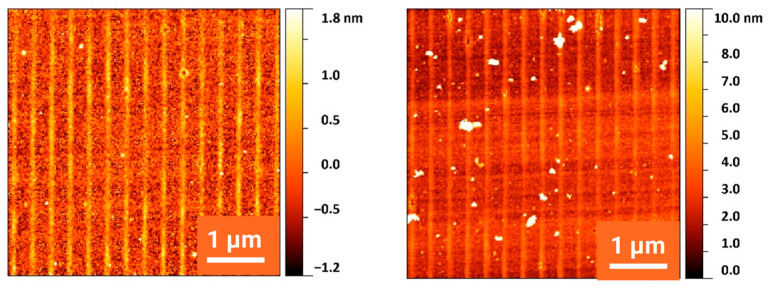
AFM record of a silicon surface irradiated at 27 mJ/cm^2^ with 1 pulse (**left**) and with 10 pulses (**right**). The height amplitude increases from 1 nm (1 pulse) to about 2 nm (10 pulses).

**Figure 5:**
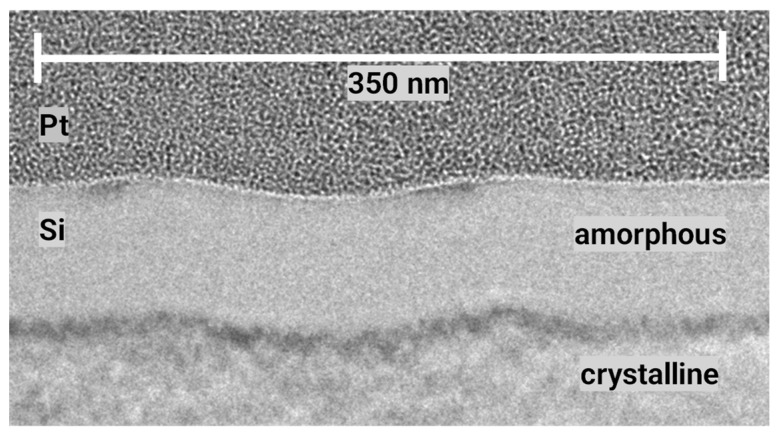
TEM cross section of a silicon surface irradiated with a single pulse at 75 mJ/cm^2^. Structure formation becomes clearly visible, and an 80 nm thick layer of amorphous silicon has formed.

**Figure 6:**
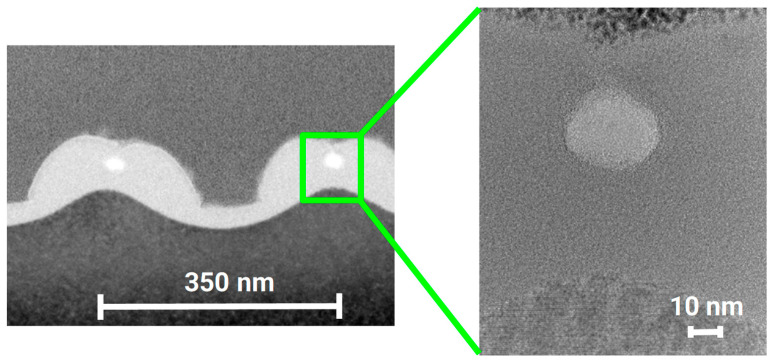
TEM cross section of a silicon surface irradiated with a single pulse at 165 mJ/cm^2^. Melt rims of neighboring grooves collide and form voids.

Figure 2 displays an overview of structures obtained at various fluences. For each fluence, an SEM top view, an AFM height profile and a TEM cross section are presented. The peak fluences in the maxima of the applied sinusoidal fluence profiles are always twice as high as the stated average fluence. At a 27 mJ/cm^2^ average fluence, the peak fluence of 54 mJ/cm^2^ is well below the multipulse ablation threshold of about 150 mJ/cm^2^ measured via large area ablation. (The single-pulse ablation threshold is not easy to determine, because the minimal changes after a single pulse near this threshold do not allow us to distinguish between material removal and material modification using standard methods.) Very shallow elevations of about 1 nm can be measured using AFM in the high-intensity regions. In these very regions, the TEM cross sections show the amorphization of the silicon material to a depth of about 30 nm (bright areas). The differentiation between amorphous (bright) and crystalline (dark) regions is confirmed by the close-up in Figure 3, where the atomic arrangement can be clearly resolved. This can be partly explained by the ~2% lower density of amorphous compared to crystalline silicon [28]. This means that at this fluence, no substantial forces for an uplift of material in the sense of a pre-ablation stage are present. This is confirmed by the observation that even after 10 pulses at this fluence, the elevation does not increase to more than 2 nm (Figure 4). At an average fluence of 75 mJ/cm^2^ (the peak fluence just reaches the multipulse ablation threshold), a completely melted and amorphous resolidified surface layer with a thickness of about 80 nm is observed (Figure 5). Obviously, at this fluence, material far beyond the intensity maximum is melted and thermal diffusion leads to a further energy transfer. Furthermore, at this fluence, a surface structure formation becomes clearly visible (AFM). Material in the peak fluence region is pushed sideways to form elevated rims, leaving behind grooves of ~10 nm in depth. At 165 mJ/cm^2^, the grooves become deeper and the rims grow, so that the rims of neighboring grooves merge, forming a rounded ridge (Figure 6). The overall height modulation increases to about 100 nm. Interestingly, below the colliding rims, a cavity of about 30 nm in diameter remains. Whereas the clearly defined boundary between the unaffected crystalline and resolidified amorphous material reflects the sinusoidal input intensity distribution, the outer surface shape is determined by the characteristics of the melt flow. Thus, buried nanochannels (or nanovoids, if the cavities are not continuous, which cannot easily be proven) with a diameter of 30 nm and 100 nm wide grooves, with an aspect ratio of about 1, can be obtained this way.

Amorphization of silicon near the surface by fs laser pulses is accomplished due to rapid cooling of the material after ultrashort heating [25]. Several authors have pointed out the importance of regarding non-thermal melting [29,30,31]. Here, it is shown that this amorphization can be controlled with a high spatial (lateral) resolution. Amorphous stripes with a width of 200 nm can be generated.

Melt expulsion is a very general phenomenon in laser material processing. In the case of periodic patterning, lateral melt movement will form sharp or rounded burrs or ridges between neighboring grooves. But also, for the nanosecond pulse irradiation of Si, melt rim formation after single-pulse irradiation has been described [32,33]. The more pronounced effect of thermal diffusion, however, then limits the pattern periods to more than one micron, and only very shallow modulation depths of less than 20 nm, even for multipulse irradiation, are obtained [34]. The lateral melt displacement combined with the formation of voids observed here looks similar, but not identical, to that found for gold under the same irradiation conditions [5]. For gold, four processes were assigned to four fluence regimes: surface swelling at low fluence of about 100 mJ/cm^2^, void formation around 150 mJ/cm^2^, wall formation at 200–250 mJ/cm^2^ and broad melting at fluences > 300 mJ/cm^2^. In the case of gold, voids develop at the positions of maximum intensity due to localized spallation, whereas in the case of silicon, voids are formed below the colliding melt rims at the positions of minimum laser intensity. Whereas gold forms sharp ridges with some droplets on top, silicon tends to form smoother shapes. 

**Figure 7:**
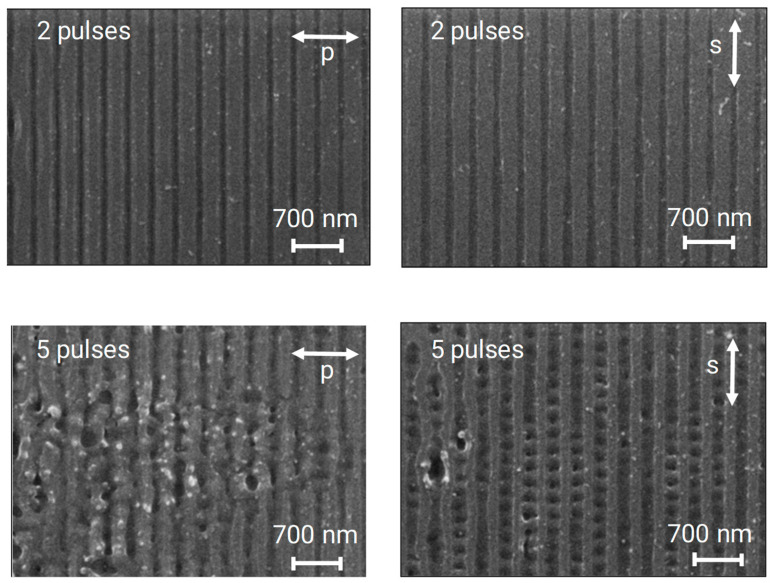
SEM top views of a silicon surface irradiated at 40 mJ/cm^2^ with 2 pulses (**top**) and 5 pulses (**bottom**). The polarization is perpendicular (**left**) and parallel (**right**) to the line pattern, respectively. A LIPSS pattern with a period of about 250 nm is seen after 5 pulses, when the polarization is parallel to the interference grooves.

It is well known that multipulse irradiation at specific fluences causes LIPSS patterns. On Si, low-spatial-frequency LIPSS (LSFL) with a period corresponding to the laser wavelength develop perpendicular to the laser polarization [11]. In Figure 7, it can be seen that this is also the case when irradiating the surface with an interference pattern instead of an unstructured beam. If the polarization is parallel to the line structure caused by two-beam interference, the LIPSS can develop unhindered with an orientation perpendicular to these lines (and to the polarization). They have, as expected, a period of about 250 nm, comparable to the laser wavelength of 248 nm. If the polarization is perpendicular to the line structure, the preferred LIPSS orientation would be parallel to this interference line structure. Since the periods of 350 nm and 250 nm do not match with each other or with one of their harmonics, both structure formation mechanisms disturb each other, and the resulting pattern becomes blurred.

Such a combination of interference and LIPSS patterns was observed in various cases. By adjusting the polarization direction, the LIPSS can either be parallel or perpendicular to the line pattern obtained via interference irradiation. If the orientation of the polarization facilitates the development of LIPSS perpendicular to the interference lines, a 2D-array of pillar-like structures can be created on stainless steel [35]. A balance between the interference structures and the LIPSS can be obtained by carefully adjusting the fluence. At low fluences, mainly LIPSS will form, whereas at high fluences, the interference structure formation will dominate. In this way, the creation of hierarchical structures with adjustable parameters can be realized, as was demonstrated via picosecond laser irradiation at 355 nm. In indium tin oxide thin films, a 75 nm LIPSS pattern was superimposed on an interference pattern with a period of 650 nm [36]. Similar patterns on stainless steel have been reported upon combining interference structures and LIPSS and applying 800 nm pulses with 100 fs duration [37].

## 4. Conclusions

Surface relief and phase change gratings on silicon are generated via UV femtosecond laser interference patterning. Periods of 350 nm and height modulation depths of 100 nm are obtained. At low fluence (27 mJ/cm^2^), 200 nm wide stripes of amorphous silicon can be generated. The amorphization depth at a fluence near the ablation threshold amounts to 80 nm. At this fluence (75 mJ/cm^2^), a surface relief develops with shallow grooves at the high-intensity positions and protrusions on both sides of the grooves. At higher fluences, lateral melt flow plays an important role in structure formation. The side walls of neighboring grooves collide, so that ridges with nano-sized voids beneath can be deterministically fabricated. After the irradiation of several pulses, a LIPSS pattern evolves, leading to a combined interference/LIPSS pattern. The results may be important for applications concerning the modification of tribological characteristics, wettability or cell growth properties, or for the generation of surface waveguides in photonic integrated circuits.

## Data Availability

The data presented in this study are available on request from the corresponding author.
